# Clinical Practice Guidelines for the Diagnosis and Management of Hereditary Fructose Intolerance

**DOI:** 10.3390/diseases12030044

**Published:** 2024-02-23

**Authors:** Félix Úbeda, Sonia Santander, María José Luesma

**Affiliations:** 1 Department of Human Anatomy and Histology, University of Zaragoza, 50009 Zaragoza, Spain; 756006@unizar.es (F.Ú.); mjluesma@unizar.es (M.J.L.); 2 Department of Pharmacology, Physiology, Legal and Forensic Medicine, University of Zaragoza, 50009 Zaragoza, Spain

**Keywords:** hereditary fructose intolerance, hypoglycemia, Fanconi syndrome, fatty liver, irritable bowel syndrome, growth retardation

## Abstract

Introduction: Hereditary fructose intolerance or hereditary fructosemia is an autosomal recessive metabolic disorder caused by a loss of function in the aldolase B gene. This disorder affects 1 in 20,000 people, constituting a rare disease with a favorable prognosis through adherence to a fructose-free diet. Despite dietary management, chronic pathology may manifest, underscoring the importance of early diagnosis to mitigate adverse effects. However, early detection of the disease poses significant challenges. Aim: Our aim was to compile pertinent information on the differential diagnosis of this pathology based on patient symptoms, facilitating the development of a diagnostic algorithm for early identification. Methodology: A systematic review adhering to PRISMA guidelines was conducted on empirical studies from PubMed, encompassing a total of 35 studies. Results: Individuals with fructose intolerance may acutely experience postprandial symptoms such as hypoglycemia, vomiting, and abdominal distension. Despite proper treatment, chronic complications such as fatty liver, Fanconi syndrome, growth deficiency, and irritable bowel syndrome may arise. The proposed diagnostic algorithm aims to minimize these adverse processes. Conclusions: Understanding the pathogenesis enables prompt diagnosis and prevention of chronicity. Establishing continuity of care from pediatric to adult medicine is crucial, and disseminating information to non-pediatric endocrinologists is imperative for managing this rare disease.

## 1. Introduction

Fructose is a monosaccharide and primarily found in fruits and some vegetables. It is commonly consumed as sucrose, a disaccharide comprising 50% fructose and 50% glucose, or as high-fructose corn syrup (42–55% fructose and the remainder glucose). Corn syrup and sucrose are prevalent sweeteners in various processed foods and beverages. The shift to corn syrup is attributed to its affordability, improved flavor, and extended shelf life [1]. Disorders related to fructose metabolism include essential fructosuria without clinical consequences and hereditary fructose intolerance or hereditary fructosemia (HFI) [2]. 

HFI is a congenital error in fructose metabolism, an autosomal recessive metabolic disease resulting from a deficiency in aldolase B gene function [3]. First described in 1956, HFI is characterized by a “functional deficiency of fructose-1-aldolase activity” [4]. HFI manifests clinically upon the introduction of fructose into the diet [5]. Variability exists in permitted foods among different diets, with prohibited items including beet, cane or maple sugar, honey, and all fruits. Glucose and dextrin-maltose are allowable, along with certain vegetables [6,7]. As patients transition from pediatric to adult care, continuity and information dissemination to non-pediatric endocrinologists are crucial. Pregnancy necessitates careful monitoring [7].

### 1.1. Epidemiology

**Figure 1:**
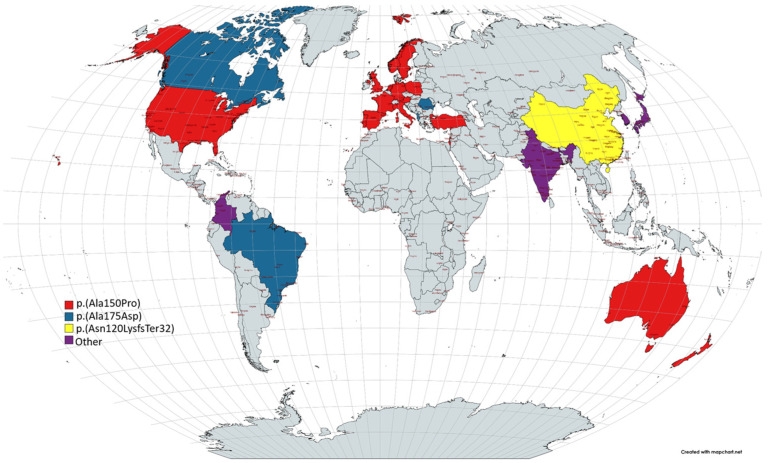
Distribution of the main variants of HFI. Based on Pinheiro et al. (2021) [8].

HFI has an estimated prevalence of 1 in 10,000 [8], although other studies suggest around 1 in 20,000 [4,7,9]. As a rare disease, its prevalence is less than 5 per 10,000 people, as defined by the European Parliament and the Council of the European Union [10]. The ALDOB gene is mapped to chromosome 9q22.3 and exhibits over 40 documented causative mutations [11]. The p.(Ala150Pro) and p.(Ala175Asp) variants are globally distributed, with distinct patterns in Europe (Figure 1) [8].

### 1.2. Pathophysiology

HFI results from aldolase B deficiency, expressed in the liver, small intestine, and kidney. This leads to fructose 1-phosphate (Fru 1P) accumulation. Endogenously produced fructose from sorbitol contributes to Fru 1P accumulation [4]. 

In a physiological context, when fructose is consumed under normal circumstances, fructokinase (KHK) initiates its conversion to fructose 1-phosphate (Fru 1P). Subsequently, aldolase B catalyzes the transformation of Fru 1P into the trioses D-glyceraldehyde and dihydroxyacetone phosphate. These intermediary trioses are then directly integrated into the glycolytic pathway, eventually giving rise to lactate and pyruvate, which serve as substrates for the Krebs cycle. Alternatively, they can be channeled into the lipogenic pathway or the gluconeogenic pathway, where they contribute to the synthesis of glucose or glycogen [9,12]. The enzymatic defect of hereditary fructose intolerance lies in aldolase B [13].

Aldolase B also catalyzes the conversion of fructose 1,6 bisphosphate (Fru 1,6-P2) to triose phosphates (dihydroxyacetone phosphate (DHAP), glyceraldehyde 3 phosphate (G3P)). In aldolase B deficiency, Fru 1P catabolism is impaired and Fru 1,6-P2 metabolism is blocked [4]. Aldolase B activity for fructose 1-phosphate is greater than for fructose 1–6 bisphosphate [13].

Most of ingested fructose is initially metabolized by the small intestine, where it is absorbed thanks to the glucose transporter 5 (GLUT5). The small intestine expresses enzymes similar to those of the liver, so fructose metabolism is similar [1,14].

Aldolase B deficiency leads to an accumulation of fructose 1-phosphate, producing liver and kidney toxicity. Glycolysis and gluconeogenesis are downregulated due to inhibition by dihydroxyacetone phosphate (DHAP) and glyceraldehyde. Additionally, adenosine triphosphate (ATP) is depleted as phosphorylation continues, blocking processes that require ATP [15]. The reduced phosphate concentration activates adenosine deaminase (ADA), resulting in the degradation of purine nucleotides to uric acid, producing the hyperuricemic characteristic of HFI [9]. Heterozygous subjects seem more susceptible to hyperuricemia and even gout illness [7]. As a consequence of phosphate depletion, hypermagnesemia and hypophosphatemia will also occur [15].

Hypoglycemia in HFI arises from deficiencies in both glycogenolysis and gluconeogenesis. These deficiencies stem from the inhibitory effects of excess fructose 1-phosphate. The compromised gluconeogenesis, coupled with the activation of pyruvate kinase by fructose 1-phosphate, leads to the accumulation of Krebs cycle precursors, alanine, lactate, and pyruvate. These compounds contribute to the observed metabolic acidosis in HFI. The condition is further exacerbated by proximal renal tubular acidosis, specifically Fanconi syndrome. This syndrome manifests with aminoaciduria, phosphaturia, and bicarbonate wasting, amplifying the metabolic challenges in HFI [9].

The cause of liver tissue damage has been attributed to the accumulation of osmotic substrates (such as fructose 1-phosphate) and ATP depletion. Ultrastructurally, hepatocytes present membranous matrices, marked rarefaction of the hyaloplasm, formation of cytolysosomes, and damage to the endoplasmic reticulum [9].

### 1.3. Clinic

Excess Fru 1P inhibits gluconeogenesis, glycogenolysis, and glycolysis, causing postprandial hypoglycemia, lactic acidosis, hypophosphatemia, hyperuricemia, and liver and kidney cellular toxicity. Clinical symptoms include nausea, vomiting, sweating, lethargy, shock, dehydration, abdominal pain, hepatomegaly, proximal tubular dysfunction, hepatic steatosis, and renal failure. It seems that healthy children can have HFI. Severe liver and kidney dysfunction, seizures, coma, and death can occur if untreated [2,5,6,16,17,18,19,20].

Vomiting is a consistent finding, while hypoglycemia-related symptoms are less common [21]. The complete exclusion of toxic sugars is challenging due to hidden amounts in many foods [5]. In adulthood, limited fructose intake (<6 g daily) is tolerated [17]. Prognosis is generally favorable with weight and biological normalization after fructose elimination. Liver enlargement and steatosis may persist indefinitely. Vitamin C deficiency risk necessitates supplements [5,7,18,22,23]. Vitamin C circulating levels are also inversely associated with metabolic syndrome [24]. A protective aversion to fructose-containing foods often develops, reducing susceptibility to tooth decay [18,22]. A dislike of sweet foods represents a good index of suspicion but occurs later than vomiting or symptoms resulting from hypoglycemia. Any suspicion of fructose intolerance should lead to immediate withdrawal of sucrose and fructose from the diet. The positive impact of removing toxic substrates becomes apparent within 2 or 3 days, serving as an initial favorable aspect in the diagnostic process before specific investigations can be conducted [25].

### 1.4. Diagnosis

Classic HFI presentation is around 6 months of age upon introducing fructose-containing foods [16]. In adults, consider HFI in unexplained liver disease, irritable bowel syndrome, or fructose-induced hypoglycemia [7].

A suggestive nutritional history or indicative aspects warrant molecular diagnosis confirmation in peripheral leukocyte DNA [7,18]. Genetic analysis via polymerase chain reaction (PCR) techniques of the aldolase B gene on chromosome 9q31 aids mutation identification [26]. It is a non-invasive approach and has advantages over the main diagnostic methods that allow measuring the enzymatic activity of aldolase B, which are both oral and intravenous fructose overload and liver biopsy [26]. An advantage over enzyme measurement in liver tissue is that genetic analysis eliminates the complication of decreased secondary aldolase activity in a damaged liver [18]. 

Liver biopsies from patients with HFI show lower Fru 1P aldolase activity levels (0–15%) compared to Fru 1,6-P2 aldolase activity (5–30%). This activity increase was used for diagnosis before the introduction of genetic testing. Liver biopsy remains the only definitive diagnostic test, since many mutations that cause HFI are unknown, in addition to variants found by DNA tests with unknown consequences [4]. Despite this, the DNA test is preferred because it is a non-invasive test; and, although the measurement of aldolase B activity in a liver biopsy (or, more rarely, kidney cortex or intestine) allows confirming the diagnosis, it is not necessary if two mutated alleles have been identified [7].

The fructose loading test is now only used exceptionally, given the risk of acute hepatocellular failure [2,7,18].

Thin layer chromatography assay offers an additional diagnostic method. It can identify fructose by analyzing urine carbohydrates. Carbohydrate-deficient transferrin (CDT) assay is often abnormal in untreated HFI due to a secondary glycosylation defect from F-1-P accumulation resulting in the inhibition of phosphomannose isomerase. Despite finding this defect, gene sequencing is required for a definitive diagnosis [16]. Although HFI is treatable, it is not included in neonatal screening since there is no biochemical marker readily available for detection without exposure to fructose [16].

The diagnostic method in many cases is not unique. After a test, the diagnosis can be confirmed with more specific tests such as a liver biopsy or genetic analysis [26].

### 1.5. Treatment

Patients diagnosed with HFI should adhere to a lifelong fructose-free diet and avoid medications containing fructose to prevent poisoning [27]. The only effective treatment for HFI is a fructose- and sucrose-free diet [6,16,18]. The optimal level of restriction in people with HFI is yet to be established, as some individuals can achieve sufficient intake reduction to normalize liver and kidney function while others may experience chronic, nonspecific symptoms despite treatment [6].

A linear correlation has been observed between fructose intake and serum carbohydrate-deficient transferrin% (serum CDT%) and carbohydrate-deficient tetrasialotransferrin/transferrin ratio (TST/CDT). Serum CDT determination could serve as a valuable tool to monitor fructose, sorbitol, and sucrose intake, with a normal CDT profile considered the desired therapeutic goal for HFI patients. This profile may also help suggest the maximum tolerability of daily fructose intake for personalized dietary therapy [5].

Dietary restriction, particularly during childhood, is emphasized by Gitzelman et al. [6]. Exclusive breastfeeding or the use of sucrose-free infant formulas can achieve rigid dietary restriction in early childhood. Introducing solid foods is not discouraged, but caution should be exercised to avoid fructose-containing foods until 2 or 3 years of age when liberalization seems tolerable [6].

The absence of specific guidelines arises from discrepancies in food composition tables detailing sugar content [6]. Radically eliminating fruits, honey, most vegetables, and other foods containing fructose is recommended. Sorbitol, present in medicines and sugar-free products, should also be excluded [28]. The presence of fructose (or sucrose or sorbitol) in many common infant formulas and most over-the-counter baby medicines is poorly recognized [16]. Glucose, maltose, and starch can replace sucrose [17].

Research on molecules inhibiting fructokinase to prevent fructose 1-phosphate accumulation is ongoing. Fructokinase deficiency results in essential fructosuria. This benign metabolic disorder is not known to cause any clinical symptoms and therefore emphasizes the therapeutic potential of fructokinase inhibition [4]. This led to the identification of pyridine 12, a safe inhibitor of fructokinase with good results in experiments with rats; it is established as a possible therapeutic method [28]. From the pyrimidine molecule, a fructokinase inhibitor began to be formed for possible clinical use and PF06835919 was discovered [29].

The pharmacological inhibition of fructokinase in humans was demonstrated for the first time in a Pfizer study of 16 subjects. It should be noted that the study did not aim to evaluate the metabolic benefit of KHK inhibition but was designed to test the safety and tolerability of PF06835919. From that, experimental inhibitors like pyridine 12 and PF06835919 showed promise in preclinical studies [28,29]. The safety and tolerability of PF06835919 are under investigation in human studies, focusing on safety rather than assessing metabolic benefits [30].

While hereditary fructosemia is manageable with a fructose-free diet, associated pathologies can occur despite correct treatment. This study aimed to compile relevant information about the pathology’s differential diagnosis based on patient symptoms and to design a diagnostic algorithm for early detection.

## 2. Material and Methods

The study utilized a multi-step methodology:

1. Initial Search: A general search with the term ‘hereditary fructose intolerance’ yielded 1089 publications in PubMed. Fourteen articles were included for a comprehensive understanding, including aspects such as symptomatology, differential diagnosis, and treatment options. This step was carried out between 1 December 2022 and 31 January 2023.
2. Systematic Search: Terms related to prevalent pathologies (kidney disease, liver disease, hypoglycemia, failure to thrive) were combined with ‘hereditary fructose intolerance’ in a systematic search. Using the Boolean operators AND and OR, the most appropriate combination of terms was created to yield the best results. The combination was as follows: (hereditary fructose intolerance) AND ((kidney disease) OR (liver disease) OR (hypoglycemia) OR (failure to thrive)). A total of 346 results was obtained in PubMed. Before proceeding to the selection of articles, the inclusion and exclusion criteria were defined:
  - Inclusion criteria: Any paper related to the pathology associated with hereditary fructose intolerance in humans, including studies, reviews, case series, editorials, guidelines, etc.
  - Exclusion criteria: Unusual manifestations, diseases not related to hereditary fructose intolerance, and, finally, pathology in animals.
  A total of 230 articles was obtained after applying inclusion and exclusion criteria; 48 articles were selected for further analysis. Fifteen articles were discarded for not adding relevant information. This step was conducted between 1 February 2022 and 31 March 2023.
3. Manual Search: Based on references from the selected studies, 2 additional articles were included, bringing the total to 35 empirical articles published between 1961 and 2023. This step took place during the month of May 2023.

**Figure 2:**
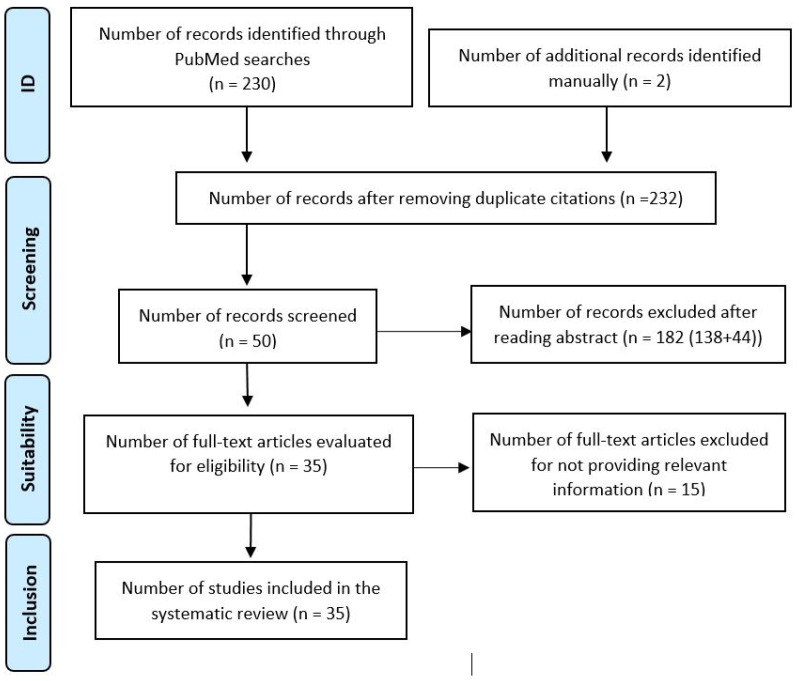
Modified PRISMA flowchart [31,32].

The study’s systematic review adhered to PRISMA guidelines, focusing on human-related pathologies associated with hereditary fructose intolerance. The following PRISMA flowchart (Figure 2) summarizes the search process. 

Two of the authors of this paper acted as independent reviewers, conducting the literature search, study selection, and assessment of methodological quality. The participation of two reviewers ensured a comprehensive review and minimized potential biases in the identification and selection of the included studies. Additionally, the third author, also a reviewer, played the role of a mediator in case of disagreements between the reviewers. 

As a tool, Mendeley (Mendeley (2022), Mendeley—Reference Management Software (Mendeley Desktop 1.19.8) & Researcher Network, https://www.mendeley.com/ (accesed on 25 January 2021)) was utilized as a bibliographic reference manager, enabling the organization and management of references from studies identified during the search. Regarding the assessment of study quality, an approach based on the authors’ criteria was followed, aligned with the 16 items specified by the AMSTAR-2 (A MeaSurement Tool to Assess Systematic Reviews) tool.

## 3. Results

The analysis of HFI reveals both acute and chronic symptoms, with varying individual sensitivity to fructose. Acute symptoms include gastrointestinal discomfort, nausea, vomiting, and hypoglycemic manifestations such as paleness, sweating, tremors, lethargy, and seizures [7].

If left undiagnosed and untreated in childhood, the pathology can persist into school age and adulthood, presenting a range of symptoms and associated pathologies, including:

1. Renal pathology:
  - Proximal tubular dysfunction.
  - Nephrolithiasis/nephrocalcinosis.
2. Growth retardation:
  - Impaired growth in children.
3. Hepatopathy:
  - Acute manifestations.
  - Fatty liver.
4. Irritable bowel syndrome.

Understanding the diverse manifestations and associated pathologies is crucial for accurate diagnosis and management of hereditary fructose intolerance. The presented information provides a foundation for further research and the development of diagnostic algorithms.

### 3.1. Hypoglycemia 

Hypoglycemia is a key, acute, adverse effect in HFI, occurring both in infancy and adulthood. It is the most common metabolic disorder in childhood, represents a diagnostic challenge, and requires an urgent therapeutic approach [18]. In HFI, the mechanism involves the accumulation of fructose 1-phosphate inhibiting glucose production via glycogenolysis and gluconeogenesis [33].

The diagnosis of hypoglycemia occurs when the blood glucose level is <0.50 g/L (<2.75 mmol/L) [34]. For neonates, the threshold is debated but may be reduced to 0.40 g/L [34]. In infants, the consideration of inborn errors of metabolism is crucial, especially when hypoglycemia occurs after the end of breastfeeding [35] when cow’s milk formulas sweetened with sucrose or foods such as fruits and vegetables are introduced [7,18].

Common causes of hypoglycemia in adults are Type 1 diabetes mellitus; certain drugs; sepsis; liver, heart, or kidney failure; cortisol deficiency; and mesenchymal tumors expressing insuline like growth factor 2 (IGF2) [18]. 

**Figure 3:**
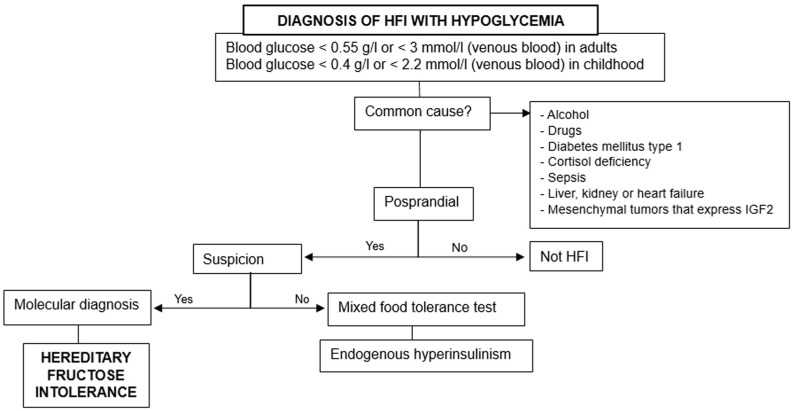
Diagnosis of hereditary fructose intolerance (HFI) with hypoglycemia (simplified diagram) [18].

Investigation steps in both children and adults are (Figure 3):

1. Rule out the previous common causes and others based on fasting, postprandial, or exercise-induced hypoglycemia.
2. Consider hereditary fructose intolerance if triggered by food ingestion.
3. Perform molecular diagnosis for confirmation, especially when there are suggestive data [18].

Exercise-induced hyperinsulinemia is related to an autosomal dominant activating mutation of the monocarboxylate transporter 1. Hypoglycemia on an empty stomach, if not due to an insulinoma, makes us think that it may be caused by an inborn error of metabolism such as storage disease of liver glycogen, defects in fatty acid oxidation, or disorders of gluconeogenesis [18,34].

Fiocchi A. et al. described the case of an infant who presented symptoms of vomiting and hypotension after eating fruit and that its withdrawal resulted in the lack of recurrence. The diagnosis was food protein-induced enterocolitis, a non-immunoglobulin E-mediated gastrointestinal food hypersensitivity that manifests as profuse, repetitive vomiting, often with diarrhea, leading to acute dehydration and lethargy. Therefore, it may be misinterpreted as HFI, and it should be considered in the differential diagnosis when there is HFI suspicion [22].

### 3.2. Kidney Pathology

Patients with HFI may experience chronic effects on the urinary system due to aldolase B deficiency in the renal cortex [35,36]. Fructose intake induces a secondary Fanconi syndrome, leading to dysfunction in the proximal renal tubule. There are clinical consequences (growth retardation; rickets; altered reabsorption of phosphate, amino acids, glucose, and uric acid [37,38,39]) that have been a cause for increased lactate and an acidification defect, characterized by a 15–30% reduction in tubular bicarbonate reabsorption [36,40].

Renal tubular acidosis (RTA) may occur [35,36,37,38,39,40,41]. It is a clinical disorder of renal acidification biochemically characterized by hyperchloremia, minimal or null azotemia, and alkaline or minimally acidic urine in the presence of metabolic acidosis [35,36]. When this syndrome is combined with reduced rates of titratable acid and ammonium excretion, it is generally considered a diagnosis of RTA [36]. To confirm the presence of proximal RTA, reduced renal bicarbonate reabsorption, increased fractional bicarbonate excretion, and failure to lower urine pH during acidemia should be confirmed [41].

Smith and Walsh described, although as a very rare condition, the appearance of nephrolithiasis in patients with Fanconi syndrome secondary to HFI; nephrolithiasis is often associated with nephrocalcinosis, which can lead to chronic renal failure [38,40].

Several studies, through the experimental induction of fructose in patients with HFI, found convincing evidence that experimental renal dysfunction induced by fructose is dose-dependent exclusive to patients with hereditary fructose intolerance and mediated by a genetic renal aldolase B deficiency [36,40,41].

Morris et al. found that circulating parathyroid hormone (PTH) could act by amplifying the rate at which the renal cortex extracts fructose and converts it to fructose 1-phosphate. Therefore, hypothyroidism would imply a lesser development of the disease since having less circulating PTH would not be able to stimulate the uptake of fructose in the renal cortex [40], which could have healing implications. 

### 3.3. Growth Delay

Children with HFI typically have normal weight and height at birth, and symptoms develop when fructose is introduced into their diet [16]. Fanconi syndrome secondary to HFI can lead to growth retardation and rickets [37]. Growth deficits may occur even in clinically asymptomatic HFI patients with an inadequate dietary fructose restriction [42]. Early diagnosis and adherence to a fructose-free diet are crucial for preventing growth delays [34].

### 3.4. Liver Pathology

Liver damage in HFI progresses from acute to chronic. Symptoms include manifestations due to hepatocellular necrosis, cholestatic states, and hepatomegaly. In patients with hepatocellular necrosis, both acute and subacute manifestations are characterized by jaundice and/or edema, ascites, bleeding tendency, and, occasionally, hepatic encephalopathy. The differential diagnosis includes severe viral hepatitis since laboratory studies can show indistinguishable results. In metabolic diseases, the liver remains enlarged, while in acute liver failure of viral origin, rapid atrophy is observed [25].

Once acute liver failure of viral origin has been discarded, some metabolic errors must be ruled out: galactosemia, fructose intolerance, and tyrosinemia type I. HFI should be considered when symptoms appear as soon as fructose is introduced in the diet. A constant finding is vomiting and, less frequently, hypoglycemia. Food aversion would start later [16,21]. On the contrary, if the patient had galactosemia, the fundamental manifestations would be jaundice and a hemorrhagic tendency. Tyrosinemia type I is considered when galactosemia and HFI have been excluded [25].

Non-alcoholic steatohepatitis (NAFLD) represents a growing epidemic worldwide and is predicted to be the leading cause of liver cirrhosis, surpassing alcohol and viral hepatitis [42]. NAFLD is currently considered the most common cause of chronic liver disease in developed countries. Traditionally, obesity and other diseases have been considered as possible etiological factors, among which are some hereditary defects of metabolism such as galactosemia, glycogenosis, abetalipoproteinemia, and fructosemia [19,43,44] in addition to other causes of liver damage such as celiac disease, Wilson’s disease, autoimmune hepatitis, or even muscle diseases [45].

Numerous studies have shown that accumulation of fructose 1-phosphate due to aldolase B deficiency produces an increase in liver fat content [4,46]. It is unclear whether small amounts of fructose can be tolerated. It has been reported that amounts up to 40 mg/kg/day or 1.5 g/day are considered safe for patients with HFI. Studies show that approximately 70% of patients had hepatic steatosis at the time of diagnosis and more than 90% at the end of follow-up [5]. We must remember to consider HFI in the differential diagnosis of children who present steatosis and liver failure, especially if the pathological findings are suggestive [9].

NAFLD is characterized by the accumulation of fat affecting >5% of the liver volume that is not explained by alcohol abuse. NAFLD can progress to cirrhosis and later to hepatocellular carcinoma; despite this, it is a benign condition, potentially reversible, and does not necessarily lead to irreversible liver damage [13,19]. NAFLD is traditionally associated with patients with hereditary fructose intolerance who have poor metabolic control and a high BMI (body mass index). Despite this, there is a high prevalence of fatty liver in patients with HFI unrelated to obesity and insulin resistance [19,47]. 

Odrieve et al. suggested that steatosis is present in young patients with HFI despite a fructose-free diet from birth. Steatosis represents a marker of metabolic alteration and persists for years and potentially for life [48]. In patients with HFI, fructose can be produced endogenously by the sorbitol-aldose reductase pathway, which can be activated in various physiological and non-physiological circumstances, for example, after a meal rich in complex carbohydrates (rice, bread, cereals), after the administration of nephrotoxic medications, during episodes of sepsis, or after cardiovascular surgery. This endogenous production of fructose could partly explain the mild signs of liver disease present in the majority of patients with HFI despite a fructose-, sucrose-, and sorbitol-free (FSS) diet [5].

During the initial stages of childhood, there are reports of giant cell hepatitis, intracanalicular cholestasis, bile duct proliferation, and the manifestation of a cirrhosis pattern within the biliary system. As childhood progresses, the prevailing characteristics shift towards macrovesicular steatosis and fibrosis. HFI may manifest later on as isolated massive hepatomegaly accompanied by steatosis in a relatively asymptomatic patient who exhibits normal liver test results and glucose levels [9].

Light microscopy in liver tissue samples from HFI patients showed diffuse and marked steatosis and fibrosis of the portal tracts. In cytoplasmic patients, it showed drops of microvesicular fat and necrosis [49].

Magnetic resonance imaging spectroscopy of the liver revealed that intrahepatic triglyceride (IHTG) content was higher in HFI [46]. The increase in hepatic triglyceride levels is partly explained because de novo lipogenesis (DNL) is greater in aldolase-deficient mice (ALDOB-KO). It was found in ALDOB-KO mice that the accumulation of fructose 1-phosphate induced the dissociation of the GKRP–GCK complex (glucokinase-glucokinase regulatory protein); GCK stimulates hepatic glucose uptake that will be transformed into glycogen and fat through DNL. Although aldolase deficiency should block fatty transformation, the pentose phosphate pathway (PPP) could be behind the conversion of glucose 6-phosphate into glyceraldehyde 3 phosphate and, thus, this transformation can occur [4].

Additional pathways accounting for the stimulation of DNL include the activation of AMP deaminase and the generation of urate, leading to mitochondrial dysfunction that, in turn, promotes DNL. Additionally, the activation of carbohydrate-responsive element binding protein (ChREBP) occurs in response to intracellular phosphate depletion. ChREBP activation stimulates the expression of glucose-6-phosphatase and genes associated with DNL [4].

Empirical investigations demonstrated that fructose 1-phosphate (Fru 1P) hinders the activity of mannose-6-phosphate isomerase (MPI), a pivotal enzyme in the glycosylation process [4]. MPI allows the passage of mannose-6-phosphate to fructose 6-phosphate [50].

Despite the limitations at the time of possible research in humans, certain advances have been described. 

The levels of beta-hydroxybutyrate in the plasma, a biomarker specifically associated with liver beta-oxidation, exhibited a notable decrease in individuals with HFI compared to their healthy counterparts. MalonylCoA, a precursor essential for the synthesis of fatty acids through de novo pathways, exerts inhibitory effects on the activity of carnitine palmitoyltransferase I (CPTI), a transporter crucial for long-chain fatty acids. Consequently, this inhibition hampers the transportation of long-chain fatty acids across the mitochondrial membrane, resulting in altered beta-oxidation. In light of these findings, it can be inferred that the biomarker profiles in HFI patients closely resemble the comprehensive phenotypic characterization observed in ALDOB-KO mice [4].

Unfortunately, it was not possible to non-invasively measure the GKRP–GCK interaction as a possible explanation for the increased triglyceride content in the liver in patients with HFI, as this required liver biopsies. However, genetic epidemiology is a good tool to predict the metabolic consequences of a GKRP-GCK alteration in humans. Rs1260326 and rs789004 are common variants in the GKRP (GCKR) gene. The first is a functional variant that encodes a GKRP protein that dissociates from GCK more easily, comparable to the effect of Fru 1P on the GKRP–GCK complex. Variants in GCKR have been associated with reduced beta-hydroxybutyrate levels, increased DNL, and increased intrahepatic triglyceride content. Furthermore, these variants have been associated with lower fasting insulin concentrations and higher glucose levels 2 h after glucose loading, the former according to ALDOB-KO mice and the latter with HFI patients [4].

### 3.5. Irritable Bowel Syndrome

The small intestine plays a crucial role in fructose metabolism, with GLUT5 facilitating fructose absorption. More than 90% of fructose is metabolized to glucose and lactate in the small intestine and exported through the portal blood to be absorbed by the liver. Limited amounts escape hepatic metabolism and enter the systemic circulation. Evidence shows that the small intestine expresses liver-like fructolytic and gluconeogenic enzymes, thus playing an important role in fructose metabolism [1].

Upon entering intestinal epithelial cells via GLUT5, fructose undergoes rapid phosphorylation to form Fru 1P catalyzed by KHK. Subsequently, Fru 1P undergoes direct cleavage into three-carbon units, DHAP, and glyceraldehyde by aldolase B. Notably, glyceraldehyde, in contrast to glycolysis aldolase products, requires phosphorylation on G3P facilitated by triokinase (TrioK). DHAP and G3P, identical to glycolytic intermediates, can enter the gluconeogenic pathway or be further metabolized through the glycolytic pathway, ultimately leading to lactate formation. Triose phosphates resulting from fructolysis can be either re-synthesized into glucose through gluconeogenesis or metabolized into lactate or acetyl-CoA, which, in turn, may be oxidized or utilized for lipogenesis [1].

The passage of unmetabolized fructose through the small intestine to the liver is dose rate dependent. Low doses of fructose are almost completely eliminated by the small intestine, but high doses of fructose saturate the intestinal fructose absorption and elimination capacity, so the extra fructose is digested by the liver and colonic microbiota [1]. 

HFI patients often experience symptoms such as abdominal pain, nausea, and vomiting after ingesting fructose due to a deficiency in fructose 1-phosphate aldolase in the cells of the small intestine [25,41]. It must be taken into account that these are most likely the initial manifestations of the disease, so it will be essential to know the context in which the symptoms have occurred [20,21]. Recurrent abdominal pain over three months may lead to the development of irritable bowel syndrome [16,21]. Self-imposed dietary restriction can alleviate gastrointestinal discomfort, but it may not prevent hepatomegaly and growth retardation, particularly in children [6].

## 4. Discussion

Understanding these diverse pathologies and their mechanisms is essential for the accurate diagnosis and effective management of hereditary fructose intolerance. The information provided offers insights into the complex interplay of fructose metabolism and its impact on various organ systems.

The difficulty in the early diagnosis of HFI is acknowledged due to the variability in symptom presentation and the potential development of protective aversions to fructose-containing foods. Early diagnosis is crucial for minimizing the consequences of the disease [21,25]. A retrospective cohort study with a large number of HFI patients could provide insights into the evolution of the disease, helping identify common alarm symptoms for early diagnosis.

Increased fructose intake exacerbates symptoms and worsens the consequences of HFI [1]. A fructose-free diet is crucial for managing the disease, but even with strict adherence, patients may develop chronic pathology. Periodic monitoring from childhood through adulthood is essential to assess the patient’s condition and adjust management accordingly [1,6,7].

The initial symptoms of HFI can overlap with multiple pathologies, emphasizing the importance of knowing when to consider HFI based on specific signs [7,16]. The study results contribute to clarifying the scenarios in which physicians should consider HFI as a potential cause of their patient’s symptoms.

**Figure 4:**
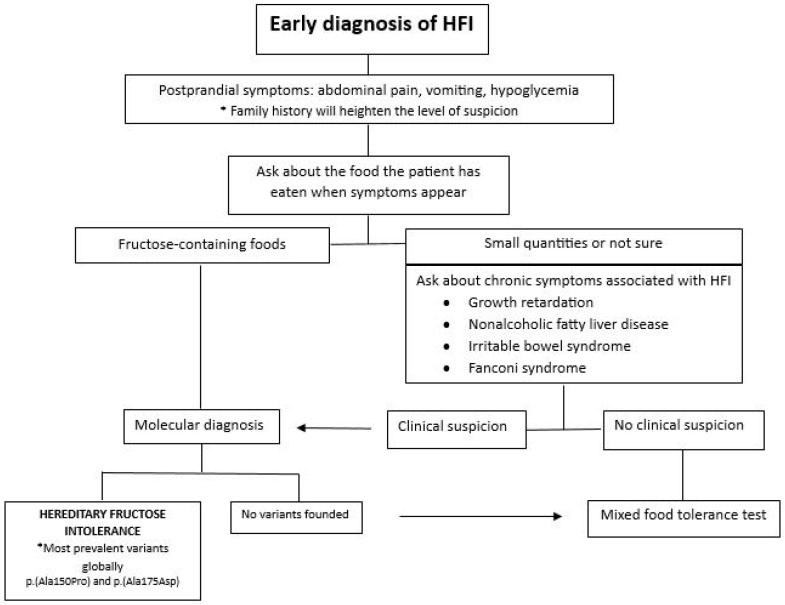
Early diagnosis scheme for hereditary fructose intolerance (HFI) in children and adults.

The development of an algorithm based on the literature review aims to assist physicians in the early diagnosis of HFI (Figure 4). 

If a patient reports experiencing abdominal pain, vomiting, and hypoglycemia following meals, in addition to an aversion to sweets and absence of cavities, which are commonly associated with the onset of symptoms, we should inquire about the specific type of food they have consumed. If the patient has ingested fructose-containing foods, we will conduct a molecular diagnosis to investigate the suspicion of HFI [5,21,25]. 

Upon identifying a mutation in the aldolase B gene, with the predominant global variants being p.(Ala150Pro) and p.(Ala175Asp), we will confirm the diagnosis of HFI. If no mutations are detected in the aldolase B gene, a mixed food tolerance test will be conducted to explore alternative pathologies. In situations where it is challenging to specify the implicated food or the fructose intake is not substantial, we will inquire about potential complications associated with HFI. If the patient has experienced growth retardation, non-alcoholic fatty liver disease, irritable bowel syndrome, hepatocellular necrosis, and/or Fanconi syndrome, suspicion of HFI will be heightened, prompting a molecular diagnosis. If clinical suspicion is not significant, a mixed food tolerance test will be employed to investigate other potential causes [5,8,37].

The discussion underscores the challenges in diagnosing HFI early, the impact of fructose intake on symptoms, the need for lifelong dietary management, and the importance of developing diagnostic tools, such as the proposed algorithm, to aid physicians in the timely identification and management of HFI. The comprehensive approach presented in this study contributes valuable insights to the understanding and diagnosis of hereditary fructose intolerance.

Health and social care needs should be taken into account in patients with hereditary fructose intolerance [51]. In addition to the proposed diagnostic algorithm, it is essential to acknowledge the significant contribution of the ‘Association of People Affected by Hereditary Fructose Intolerance’ (AAIHF). This non-profit association, composed of affected families, plays a crucial role in uniting individuals with HFI and raising awareness globally about this rare genetic disorder. 

Visit the AAIHF website at https://www.aaihf.com/index.php (accessed on 19 February 2023) for comprehensive information about the disease [52]. The association provides valuable resources, including downloadable brochures in multiple languages containing recommendations for school, a diet guide tailored for HFI patients, suggestions for dining out, and guidelines for health care professionals regarding medication excipients.

With the exclusion of fructose, sorbitol, and sucrose from the diet, the prognosis for individuals with HFI is generally favorable. The AAIHF’s resources, such as the diet guide, offer practical assistance in managing dietary restrictions. However, the impact of the disease is variable among individuals. Some patients can successfully achieve a low enough fructose intake to normalize liver and kidney function. On the other hand, there are cases where chronic pathology persists despite adherence to the treatment regimen.

Given the nonspecific impact and varying responses to treatment, ongoing research is crucial to advancing our understanding of HFI. The AAIHF’s advocacy and awareness efforts contribute significantly to garnering support for research initiatives. Despite economic challenges associated with studying rare diseases, progress is being made in exploring new possibilities for treating HFI. Recent advancements, such as pharmacological inhibitors of fructokinase and experimental studies, represent promising avenues for future therapeutic interventions.

## 5. Conclusions

Understanding the pathogenesis enables prompt diagnosis and prevention of chronicity. Establishing continuity of care from pediatric to adult medicine is crucial, and disseminating information to non-pediatric endocrinologists is imperative for managing this rare disease. 

The collaborative efforts of associations like the AAIHF, along with advancements in research, offer hope for improved diagnosis, management, and potential treatments for hereditary fructose intolerance. 

The commitment to raising awareness and facilitating information dissemination is paramount in supporting individuals and families affected by this rare genetic disorder.

## Data Availability

Data are contained within the article.

## Abbreviations

HFI (hereditary fructose intolerance), ALDOB (aldolase B), Fru 1P (fructose 1-phosphate), Fru 1,6-P2 (fructose 1,6 bisphosphate), KHK (fructokinase), DHAP (dihydroxyacetone phosphate), G3P (glyceraldehyde 3 phosphate), GLUT5 (glucose transporter 5), ATP (adenosine triphosphate), ADA (adenosine deaminase), PCR (polymerase chain reaction), CDT (carbohydrate-deficient transferrin), TST (tetrasialotransferrin), ATR (renal tubular acidosis), PTH (parathyroid hormone), NAFLD (non-alcoholic fatty liver disease), BMI (body mass index), FSS (fructose/sucrose/sorbitol), DNL (lipogenesis de novo), ALDOB-KO (aldolase deficiency), ChREBP (carbohydrate-responsive element binding protein), GKRP-GCK (glucokinase-glucokinase regulatory protein), PPP (pentose phosphate pathway), MPI (mannose-6-phosphate isomerase), CPTI (carnitine palmitoyltransferase I), TrioK (triokinase), IGF2 (insuline like growth factor 2).
